# The acute effect of exercise and nutrition on respiratory exchange ratio in women

**DOI:** 10.1186/1550-2783-11-S1-P5

**Published:** 2014-12-01

**Authors:** Hailee L Wingfield, Abbie E Smith-Ryan, Malia N Melvin, Erica J Roelofs, Eric T Trexler

**Affiliations:** 1 Applied Physiology Laboratory, The University of North Carolina, Chapel Hill, North Carolina, USA

## Background

Few studies exist evaluating metabolic responses to exercise and nutrition in women. Understanding sex-specific fuel differences may improve exercise prescription. PURPOSE: To examine the effect of exercise modality and pre-exercise carbohydrate (CHO) or protein (PRO) ingestion on respiratory exchange ratio (RER) in women.

## Methods

Twenty recreationally active women (Mean ± SD; age 24.6 ± 3.9 yrs; height 164.4 ± 6.6 cm; weight 62.7 ± 6.6 kg; %fat 28.2 ± 4.8 %) participated in this randomized crossover, double-blind study. After preliminary body composition and maximal strength testing, each participant completed six exercise sessions, consisting of three exercise modalities: aerobic endurance exercise (AEE; 30 min, 50% heart rate reserve), high-intensity interval running (HIIT; 10 x 1min on: 1min off, 90%HRR), and high-intensity resistance training (HIRT; 6 exercises: 3 sets, 20 sec rest), and two acute nutritional interventions: 25 grams of CHO (maltodextrin) and PRO (whey protein isolate). Salivary samples were collected before each exercise session to determine estrogen. RER was analyzed via indirect calorimetry (Parvomedics TrueOne 2400) at: baseline, immediately post (IP), 30 minutes (30min) post, and 60 minutes (60min) post-exercise. Subjects were seated and connected to the metabolic cart by a tube for 15 minutes at each time point. A mixed level model [modality (AEE vs. HIIT vs. HIRT) × treatment (CHO vs. PRO) × time (base vs. IP vs. 30min vs. 60min)] covaried for estrogen was used to evaluate RER. Consent to publish the results was obtained from all participants.

## Results

Significant two-way interactions existed between modality and time (p<0.0001) and time and treatment (p<0.0001). Significant modality differences existed between HIIT and AEE (p<0.0001), HIIT and HIRT (p<0.0001), and AEE and HIRT (p=0.0024). RER was significantly higher from HIIT than AEE and HIRT IP exercise (p<0.0001), and significantly lower from HIIT 30min and 60min post exercise (p<0.0001-p=0.0020) than AEE. RER was significantly lower 30min (p=0.0169), but not 60min post (p=0.3603) HIIT vs. HIRT. No significant differences existed between HIRT and AEE IP exercise (p=0.3370); RER was significantly lower for HIRT than AEE 30min and 60min post exercise (p=0.0004-0.0265). For time and treatment, RER was not significantly different between CHO and PRO IP exercise (p=0.1150), but was significantly lower for PRO 30min and 60min post (p<0.0001-p=0.0012).

## Conclusions

HIIT produced lower RER than AEE from 30 to 60min post, and a lower RER than HIRT 30min post exercise. PRO ingestion prior to exercise decreased RER more than CHO ingestion beginning 30min post exercise. HIIT and HIRT were the most effective and time-efficient approaches at increasing fat utilization after exercise. Thirty minutes post exercise, fat utilization was increased from HIIT and HIRT compared to AEE. One hour after exercise, HIIT and HIRT were similar in fat utilization. PRO ingestion prior to exercise increased fat utilization more than CHO. Combining HIIT and HIRT with pre-exercise PRO intake in women may lead to greater changes in weight and body composition due to decreased RER.

